# Supplementary material for: Lipid droplet distribution quantification method based on lipid droplet detection by constrained reinforcement learning
Source: PLoS One. 2025 Sep 25;20(9):e0332884. doi: 10.1371/journal.pone.0332884 (PMC12463257; doi:10.1371/journal.pone.0332884)
Supplement: Attachment — Submitted filename: response_for_comment__0622, final.docx [file pone.0332884.s001.docx]

1. Lack of Quantitative Validation of the Detection Algorithm

The manuscript does not provide objective performance metrics for the revised LiDRL method. Although expert agreement is mentioned, no numerical accuracy metrics are reported.

Annotate a subset of your image dataset with expert-labeled ground truth and evaluate model performance using standard detection metrics (e.g., sensitivity, specificity, precision, recall, F1 score). Include a description of how these metrics were calculated, and if possible, inter-rater reliability between experts.

Thank you for your comment. In addition to the results of Experiment 3, we have added the following metrics: “Performance evaluation of the conventional method and the proposed method” and “Calculation method,” which are summarized in Table 4. Changes were made, as follows:

# “Experiment 3: Comparison between the conventional LiDRL method and the revised LiDRL method

We compared the performance of the conventional and revised LiDRL methods, leading us to modify the experimental and calculation protocols accordingly. The performance of both models was evaluated using sensitivity, specificity, accuracy, precision, recall, F1 score, and CV (Equations 3 to 9) values, obtained over an average of five runs. In addition, as the performance evaluation of the conventional LiDRL method [21] used the F1 score based on 10-fold cross-validation, the same comparison criteria were used for the revised LiDRL method.

Sensitivity= TP / (TP + FN) (3)

Specificity= TN / (TN + FP) (4)

Accuracy = (TP + TN) / (TP + TN + FP + FN) (5)

These measures represent the overall correctness of the classification model.

Precision = TP / (TP + FP) (6)

It evaluates the proportion of predicted positives that are truly positive.

Recall = TP / (TP + FN) (7)

It indicates the ability of the model to accurately capture true positives.

F1 score = 2 × Precision × Recall / (Precision + Recall) (8)

It balances precision and recall, which is useful when classes are imbalanced.

Cross-validation F1-score =$\frac{1}{k}\sum_{i = 1}^{k} {F1}_{i}$ (*k* = 10) (9)

TP (True Positive): Correctly predicted positives

TN (True Negative): Correctly predicted negatives

FP (False Positive): Incorrectly predicted positives

FN (False Negative): Incorrectly predicted negatives

Furthermore, to compare and evaluate the performance of the revised method versus the conventional method, two pathologists identified lipid droplets on the same images.”

**Table 4. Performance evaluation of the Conventional LiDRL method and the Revised LiDRL method.**

| **Image** | **Sensitivity** | | **Specificity** | | **Accuracy** | | **Precision** | | **Recall** | | **F1-score** | | **CV** | |
| --- | --- | --- | --- | --- | --- | --- | --- | --- | --- | --- | --- | --- | --- | --- |
|  | Con | Rev | Con | Rev | Con | Rev | Con | Rev | Con | Rev | Con | Rev | Con | Rev |
| Image001 | 0.95 | 0.91 | 0.84 | 0.72 | 0.895 | 0.815 | 0.861 | 0.765 | 0.95 | 0.91 | 0.902 | 0.831 | 0.891 | 0.914 |
| Image002 | 0.91 | 0.94 | 0.88 | 0.86 | 0.895 | 0.9 | 0.886 | 0.872 | 0.91 | 0.94 | 0.896 | 0.904 | 0.899 | 0.893 |
| Image003 | 0.9 | 0.89 | 0.82 | 0.87 | 0.86 | 0.88 | 0.834 | 0.874 | 0.9 | 0.89 | 0.865 | 0.881 | 0.907 | 0.899 |
| Image004 | 0.87 | 0.89 | 0.85 | 0.85 | 0.86 | 0.87 | 0.854 | 0.857 | 0.87 | 0.89 | 0.861 | 0.872 | 0.905 | 0.896 |
| Image005 | 0.92 | 0.93 | 0.84 | 0.86 | 0.88 | 0.895 | 0.857 | 0.874 | 0.92 | 0.93 | 0.884 | 0.899 | 0.898 | 0.899 |
| Image006 | 0.87 | 0.87 | 0.9 | 0.87 | 0.885 | 0.87 | 0.898 | 0.873 | 0.97 | 0.87 | 0.881 | 0.869 | 0.897 | 0.899 |

*Con; Conventional LiDRL method, Rev; Revised LiDRL method, CV; Cross-validation F1-score

We have also revised the discussion section to reflect these changes. The discussion was revised as follows:

“ In this study, we improved the dynamic management of the behavior of the agent and obstacle in order to assess the overall trend of lipid droplet distribution. This facilitated reinforcement learning and increased the search for combination patterns at each rank level. Consequently, it improved the ability to capture the distribution of lipid droplets of similar size and symmetry, thereby improving practical efficiency (Experiment 3). Although the revised method used a dataset different from that used by the conventional method, it achieved an F1 score within the acceptable range (0.9 ± 0.05). The precision and recall were preserved regardless of the F1 score of 0.85, which suffices in practice (Table 4). Nevertheless, the revised method demonstrated similar performance to the conventional method in terms of sensitivity, specificity, accuracy, precision, recall, F1 score, and CV. Although the accuracy of the model may be enhanced in future studies through the utilization of larger training datasets, the primary objective of our study was to train the model on a small dataset.”

In this study, we did not compare our method with existing supervised learning models such as CNN and U-Net. These models involve image classification inaccuracy and overfitting risk, and make it difficult to preserve segmentation. To clarify this point, have reorganized the references [5-20] and added the following information to the introduction:

“To address this, several methods using neural networks have been proposed [5-14]. Advanced techniques, such as Convolutional Neural Network are suitable for image classification but have limitations for preserving fine structures such as segmentation [15-17]. A method using U-net has been proposed; however, it involves a risk of increased computational complexity, memory requirements, and overfitting due to the symmetric structure of the encoder and decoder [18-20]. These methods were deemed unsuitable for this study and therefore were not utilized.”

In addition, to compare the present and conventional method, the numbers of lipid droplets identified by two pathologists on the same images are presented in Table 5.

**Table 5. Comparison of correct answer rates between conventional and revised methods.**

|  | Image A | | Image B | |
| --- | --- | --- | --- | --- |
| Hierarchy | Con | Rev | Con | Rev |
| Rank6 | Not detected | Not detected | 77.8% (21/27) | 84.0% (21/25) |
| Rank5 | Not detected | 100% (5/5) | 95.2% (20/21) | 95.5% (21/22) |
| Rank4 | 92.8% (154/166) | 92.3% (144/156) | 86.4% (248/287) | 86.8% (244/281) |
| Rank3 | 89.5% (450/503) | 88.8% (437/492) | 88.4% (281/318) | 87.9% (270/307) |

*Con; Conventional LiDRL method, Rev; Revised LiDRL method

*The numbers in parentheses are the number of correct /detections lipid droplets

We revised the discussion section to reflect these changes:

“Using two images from the same study [21], we compared the detection capabilities of the conventional and revised methods (Table 5). First, in contrast to the revised method, which classified the droplets by size, the conventional method failed to classify medium-sized lipid droplets into Rank 5. The percentage of correct answers obtained from the evaluation of the detected lipid droplets by the two pathologists showed little difference between the two methods. Similar to the conventional method, the revised method occasionally misclassified sinusoids as lipid droplets when assessing them as potential small droplets. Future studies should aim to improve the accuracy of discriminative learning.”

2. Insufficient Sample Size and Lack of Dataset Description

Only six images are used, but there is no information on whether these represent different patients or anatomical zones.

Clearly describe the dataset: how many patients it includes, whether different liver regions were imaged, and how these images were selected. Discuss how the small sample size limits the generalizability of your conclusions.

Thank you for your comment. We have added the following information to the methods section:

“In this study, we used six images, with two images taken from each of three different patients. These included the central and portal veins (each image size: 1600 × 1200 pixels, grayscale: color, 8 bits). The images were randomly selected based on anatomical requirements (one portal vein region, one central vein region). However, the results of an analysis using a limited number of images may have limited applicability when extrapolated to the entire patient population. Therefore, we estimated the shape of the distribution using KDE plots and quantified the randomness and information content of the dataset using entropy analysis. Using KDE, we effectively estimated the shape and pattern of data distribution even with a limited number of images, thus facilitating more accurate feature extraction. By evaluating the complexity of the information (data diversity and bias) using entropy analysis, we obtained the information required for suitable model selection in the next analysis.”

We have added the following to the discussion section:

“This study was designed to be generalizable even with a small number of images; however, the small sample size may have affected the generalizability of these results. Therefore, we used the KDE plots and entropy analysis to improve generalizability.”

3. Non-Compliant Data and Code Availability

Your current Data Availability Statement states that data will be made available after acceptance. This is not compliant with PLOS ONE’s policy, which requires all relevant data and code to be available upon submission.

Deposit the source code (for the revised LiDRL algorithm, KDE, and entropy tools) and a representative image dataset or simulated images in a public repository (e.g., GitHub, Zenodo, Figshare). Update your Data Availability Statement to include links or DOIs.

Failure to provide access to code and data prevents other researchers from verifying or building upon your work.

Thank you for your comment. We have made the required information available as "Revised LiDRL Algorithm, KDE, and Entropy Analysis" using Google Collaboratory.

<https://zenodo.org/records/15733729>

4. Missing Statistical Comparisons and Uncertainty Measures

Your results present averages for entropy and droplet counts, but no statistical tests or confidence intervals are included.

Conduct and report appropriate statistical comparisons (e.g., ANOVA, Mann–Whitney U test) to determine whether differences across ranks or anatomical zones are significant. Include p-values, standard errors, or confidence intervals.

Thank you for your comment. We have added the following information in the methods section (Experiment 2):

“The average values obtained from the six images were evaluated using the Mann–Whitney U test, p-value, and standard error to determine whether there were significant differences between the anatomical regions.”

Table 3 was added to the results section:

**Table 3. Variations between the central vein region and the portal vein region [six images]**

| **Category** | **Hierarchy** | **Central vein region** | **Portal vein region** | **Mann-Whitney U** | **p-value** | **Relative Error [%]** |
| --- | --- | --- | --- | --- | --- | --- |
| **Lipid Droplets Count** | Rank 6 | 36.0 | 23.3 | 7.0000 | 0.4000 | 35.19 |
|  | Rank 5 | 55.7 | 12.7 | 6.0000 | 0.5050 | 6.59 |
|  | Rank 4 | 241.0 | 256.0 | 4.0000 | 1.0000 | 6.22 |
|  | Rank 3 | 283.3 | 220.3 | 8.0000 | 0.2000 | 22.24 |
| **Average Probability Density** | Rank 6 | 0.0186 | 0.0296 | 2.0000 | 0.4000 | 59.29 |
|  | Rank 5 | 0.0115 | 0.0637 | 0.0000 | 0.0765 | 452.13 |
|  | Rank 4 | 0.0027 | 0.0026 | 5.0000 | 1.0000 | 3.80 |
|  | Rank 3 | 0.0023 | 0.0030 | 1.0000 | 0.2000 | 33.57 |
| **Average Entropy** | Rank 6 | 2.557 | 2.268 | 7.0000 | 0.4000 | 11.29 |
|  | Rank 5 | 2.844 | 1.831 | 9.0000 | 0.0765 | 35.62 |
|  | Rank 4 | 3.777 | 3.808 | 4.0000 | 1.0000 | 0.84 |
|  | Rank 3 | 3.881 | 3.709 | 8.0000 | 0.2000 | 4.44 |

We have also added information to the discussion section, as follows:

“The number of lipid droplets in the histological regions, the central and portal vein regions, were comparable across all ranks. Regarding the relative error, the difference was small for Rank 5 and Rank 4, suggesting that the differences between anatomical regions were relatively small. Meanwhile, the error was slightly larger for Rank 6 and Rank 3, suggesting that the patterns between anatomical regions may differ depending on the rank.

In addition, the relative error for rank 5 when using the average probability density showed a stronger tendency than the other ranks. The dataset may have included large variability or outliers. No significant differences were observed for the other ranks (Ranks 6, 4, and 3). No clear differences were observed between anatomical regions. Despite a limited number of images used, the average entropy for Rank 5 indicates differences among anatomical regions. Meanwhile, Rank 4 revealed no differences, suggesting comparable entropy between anatomical regions. There were no significant differences between anatomical regions in other ranks.”

We have added the following to the discussion section:

“In addition, as the difference between the portal and central vein regions may be associated with the underlying diseases, our objective is to conduct a more extensive study in the future, using comparisons between ranks, and entropy analysis.”

5. Incomplete Biological Interpretation of Findings

The manuscript does not clearly explain what clinical or pathological insights are gained from the lipid droplet distribution patterns you identify.

Expand the Discussion to explain how differences in droplet patterns (entropy, KDE) relate to known liver disease processes, such as steatosis, fibrosis, or NASH. Discuss how your method could assist in diagnosis or research.

We have added the following to the discussion section:

“Lipid droplet analysis is not currently used in the histopathological diagnosis of fatty liver disease; conventionally, qualitative evaluation was mainly performed to confirm the presence or absence and amount of lipid droplets. However, the average probability density and entropy methods used in this study enables the quantification of droplet characteristics such as symmetry and distribution. In our future studies, we aim to evaluate the correlation between droplet characteristics and known liver diseases such as fatty liver, fibrosis, and NASH.”

6. Language Clarity and Technical Terminology

Some terminology is inconsistently used, and grammatical issues affect clarity. Terms like “rank,” “penalty area,” and “agent” should be defined and used consistently.

Please revise the manuscript for grammar and clarity, ideally using professional language editing services. Ensure all technical terms are defined when first introduced.

The manuscript was edited by a professional medical editing service.

We have included the relevant definitions in Fig. 1.

“We used the following terms to explain our algorithm: filter size, a square frame with one side as large as the maximum diameter that can contain only one lipid droplet; rank, order within a 3 × 3 filter region in a binary image; environment, a 4 × 10 grid; reward, the ratio of overlapping region to detected lipid droplet region (Fig 1).

**Fig 1. Definition of reward: Overlapping region of the newly extracted region ([21]).**

agent, a subject that moves, learns, and selects actions that maximize rewards based on the state of the environment; state, the position where the agent is in the environment. It was defined as a filter size and rank; action, the movement the agent can choose in each state. The agent can move leftward or downward; Q value, the total reward expected from a specific action in a certain state; penalty area, if the agent enters this area, a negative reward is imposed, and the agent is forced to return to the starting point. This area maintains accuracy by repeating a certain number of movements; start, the initial position of the agent; goal, the final position the agent reaches.”

7. Missing Ethics Statement in the Main Text

The ethics statement appears only in metadata, not in the Methods section.

Add the following to your Methods section:

“Experiment images were acquired in accordance with ethical research standards (Oita University Faculty of Medicine Ethics approval no. 2568/15 June 2023). The Medical Ethics Review Board approved a waiver of informed consent for this retrospective study.”

Thank you for your comment. We have included an ethics statement in the manuscript.

8. Figure Quality and Descriptions

Figures were not accessible during editorial review, and current captions are not fully descriptive.

Upload all figures as high-resolution files (TIFF/PNG). Revise figure legends to ensure they describe the image content fully, including any color scales, segmentation overlays, and reference annotations.

Thank you for your comment. We have made these changes, as required.
