# Supplementary material for: Lipid droplet distribution quantification method based on lipid droplet detection by constrained reinforcement learning
Source: PLoS One. 2025 Sep 25;20(9):e0332884. doi: 10.1371/journal.pone.0332884 (PMC12463257; doi:10.1371/journal.pone.0332884)
Supplement: Attachment — Submitted filename: Answer_to_the_Review_Comments0812.docx [file pone.0332884.s002.docx]

Reviewer #1: I suggest that the authors carefully correct minor typographical errors and ensure that all plots and figures are properly arranged before final submission.

Thank you for your comments. We have proofread the whole document again and made the necessary corrections.

Reviewer #2: The low number of training and validation samples remains a limitation of this study. While it would have been preferable to expand the number of images in both the training and especially the validation dataset, the authors now acknowledge and discuss the limitations arising from this issue in the revised manuscript. This is appreciated and can be regarded as having been adequately addressed.

Thank you for your constructive comments. As you pointed out, we also recognize the small number of cases as a limitation. The following comments have been added and corrected in the discussion on page 17: “The main purpose of this study was to train a model with a small dataset. Given the difficulty in improving accuracy through dataset expansion, future research should explore alternative algorithms beyond random forests.” It remains a challenge, and we plan to continue our research on this topic in the future.

A minor correction is recommended in the legend of the revised Table 5. The phrase "number of correct /detections lipid droplets" likely intends to mean "correct/detected lipid droplets". More importantly, expressing this ratio as detected/correct would be clearer, especially since the method appears to over-detect lipid droplets.

The legend in Table 5 has been revised to read, "The numbers in parentheses are the number of detected/correct lipid droplets." The numbers in Table 5 have also been changed accordingly.

Lastly, in response to reviewer comment 1 and the updated Table 5, the authors note that two pathologists’ annotations were used for comparison. It would be helpful if the authors could also state the total number of lipid droplets identified by the two pathologists (or their average, if the counts differ) on the validation images independent of the hierarchic rank of the employed analysis method. This would give readers a better understanding of the clinical relevance of the proposed method, independent of the ranking process.

The number of lipid droplets that two doctors simultaneously identified is shown on the verification image. Additionally, the following comment has been added to the discussion on p.18: "A detected-to-correct lipid droplet ratio greater than 1 indicates over-detection. Therefore, we used the F-score for evaluation. The correct number of lipid droplets is the same number of lipid droplets identified independently by both pathologists."
